# Sweet Syndrome Associated with Upper Respiratory Infection and Amoxicillin Use

**DOI:** 10.7759/cureus.568

**Published:** 2016-04-12

**Authors:** Mark Volpe

**Affiliations:** 1 School of Medicine, Yale University

**Keywords:** sweet syndrome

## Abstract

Sweet syndrome (acute febrile neutrophilic dermatosis) is an uncommon dermatologic eruption characterized by acute onset of painful papules, plaques or nodules on the skin that are red, blue, or violaceous in color. It has been associated with various infections, medications, and malignancies. Here we report the case of a middle-aged male who presents with Sweet syndrome after an upper resipiratory infection and while using amoxicillin. We also review the diagnostic criteria, laboratory testing, and treatment options.

## Introduction

Sweet syndrome (acute febrile neutrophilic dermatosis) is an uncommon dermatologic eruption characterized by acute onset of painful papules, plaques or nodules on the skin that are red, blue, or violaceous in color. To date, the pathogenesis is largely not understood. Typically the rash is accompanied by fevers and elevated inflammatory markers including leukocytosis, erythrocyte sedimentation rate (ESR), C-reactive protein (CRP), and neutrophilia. Though rare, extracutaneous manifestations may include involvement of the eyes, musculoskeletal system or internal organs.

The condition was first reported in 1964 [1] and since then has been associated with several disorders, prompting the medical community to divide the etiology of Sweet syndrome into three major categories. The majority of cases are classified as *classical Sweet syndrome*, usually appearing one to three weeks after infection, with inflammatory bowel disease, or with pregnancy. *malignancy-associated Sweet syndrome* represents approximately 20% of cases and is associated with several cancers, most commonly acute myelogenous leukemia and other blood cancers. Finally, *drug-induced Sweet syndrome* usually develops one to two weeks after exposure to an inciting drug. To date several classes of medications have been associated with Sweet syndrome including antibiotics, antiepileptics, antivirals, antihypertensives, non-steroidal anti-inflammatory drugs, and most prominently colony-stimulating factors. 

## Case presentation

A 41-year-old Caucasian male with past medical history of anxiety and gastroesophageal reflux disease presented to the primary care clinic with a four-day history of rash on his left hand, right forearm, and right forehead. Two weeks prior he was diagnosed with an upper respiratory infection. Three days prior to onset of the rash he presented to the clinic with tonsillitis and was started on amoxicillin 875 milligrams twice daily for seven days. He reported the rash on his left hand and right forearm to be purple and painful. One of the lesions was weeping. His right forehead rash was not painful, but was purple and looked to him like large pimples. Since he noticed the lesions, they had not significantly changed or spread to other areas of his body. He reported no new soaps, lotions, detergents, environmental exposures, newly started medications aside from the amoxicillin, foods, or history of other skin problems. He had tried applying antibiotic ointment to the lesions, thinking they were infected, without any benefit. No other treatments were attempted. He denied fevers, chills, chest pain, shortness of breath, nausea, vomiting, diarrhea, abdominal pain, joint pain, weakness, fatigue. He endorsed sore throat and occasional dry cough.

**Figure 1:**
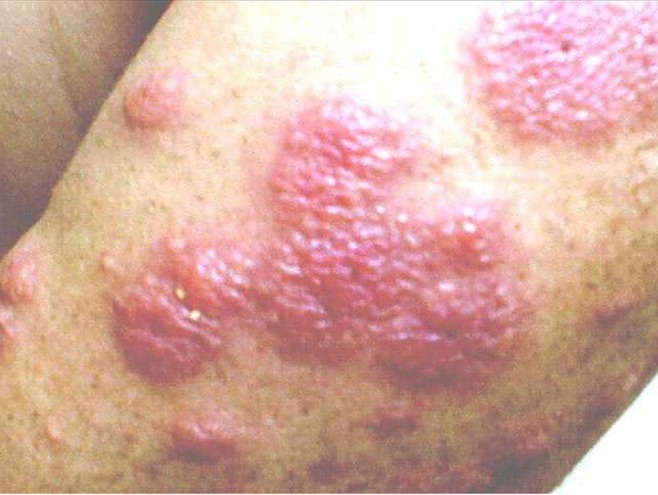
Appearance of Skin Eruption

On physical exam his blood pressure was 120/72, heart rate 84, respiratory rate 12, and temperature 98.4 degrees Fahrenheit. He was well-developed, well-nourished. His left hand had four, 1–3 cm centimeter dark purple plaques, one of which was bullous and weeping clear exudate. On his right forearm there were two 1-cm purple plaques (Figure 1). On his right forehead there were seven 5-mm purple papules. His oropharynx was erythematous with enlarged tonsils. His ears were clear bilaterally. No cervical lymphadenopathy was found. His cardiovascular, pulmonary, and abdominal exam were all within normal limits.

At this time the differential diagnosis was broad including viral exanthema, drug-eruptions, drug-induced lupus, vasculitis, and hematologic malignancy. A complete blood count with manual differential, ESR, CRP, urinalysis, and basic metabolic panel were ordered. Bacterial and viral cultures were taken from the weeping lesion. Additionally, a 4-mm punch biopsy from the left hand was acquired and sent to pathology. The patient was told to stop taking the amoxicillin and follow up in one week.

Initial lab work revealed a white blood cell count of 15.4 x 10^9^/L with 70.5% neutrophils. ESR and CRP were within normal limits. Smear revealed no hematologic malignancy. There were no other noteworthy findings. Bacterial and viral cultures were negative. Punch biopsy results revealed neutrophilic dermatosis without vasculitis, most consistent with Sweet syndrome. Gram and periodic acid Schiff with diastase stains did not find any microbial pathogens.

The patient was started on a tapered course of prednisone for four weeks. The rash resolved within the first week and has since not returned after finishing the course of steroids. Informed consent was obtained from the patient for this study.

## Discussion

Here we report a case of Sweet syndrome associated with upper respiratory infection and amoxicillin use with several unique features. Firstly, our patient was afebrile. Even during the initial presentation of the upper respiratory infection the patient presented without a fever. Of note, the patient was not taking antipyretics, but was taking amoxicillin prior to the rash onset and when his temperature was taken in our office. Typically, patients with Sweet syndrome present with fever.

Additionally, though the most likely cause of the outbreak was the upper respiratory infection that began two weeks prior, it was important to rule out the most common hematologic malignancies associated with this condition through blood work and manual smear. Furthermore, though less likely, it is possible that the rash could have been triggered by amoxicillin use. Though these rashes typically begin one to two weeks after initiating a new drug, we cannot completely rule out amoxicillin as the culprit. Several other antibiotics including minocycline, nitrofurantoin, norfloxacin, ofloxacin, and trimethoprim-sulfamethoxazole have been associated with Sweet syndrome. Typically, drug-induced Sweet syndrome will occur upon re-exposure to the drug. Thus, this would likely be the best way to rule out amoxicillin as the cause [2].

Approximately 80% of adults who develop classical Sweet syndrome are females, while 70% of adults who develop drug-induced Sweet syndrome are females [2]. Diagnostic criteria include both major and minor criteria. Patients must meet both major criteria and two of four minor criteria to confirm the diagnosis. Major criteria include (1) abrupt onset of painful, erythematous plaques or nodules and (2) histopathologic evidence of a dense neutrophilic infiltrate without evidence of leukocytoclastic vasculitis. Minor criteria include (1) Fever > 38 degrees Celsius; (2) Association with underlying hematologic or visceral malignancy, inflammatory disease or pregnancy, or preceded by upper respiratory infection, gastrointestinal infection or vaccination; (3) excellent response to treatment with systemic glucocorticoids or potassium iodide; and (4) three of four abnormal laboratory values including elevated ESR, positive CRP, >8000 leukocytes, and > 70% neutrophils [3].

Our patient responded to the first-line therapy, oral steroids, as expected with resolution of the rash within one to two weeks. Alternative options supported through case reports include topical corticosteroid therapy [4], colchicine [5], dapsone [4], and potassium iodide [6]. These options are generally considered for patients who fail oral steroids, are unable to tolerate oral steroids, or have a contraindication to their use.

## Conclusions

Sweet syndrome, though rare, is an important diagnosis to be recognized among primary care providers and dermatologists. A high level of suspicion is needed in any patients who present with the characteristic findings, and evaluation must include laboratory testing and skin biopsy. Regardless of whether or not the syndrome was preceded by infection or medication use, the most common malignancies should be ruled out. Diagnostic criteria exist to aid the clinician in confirming the diagnosis, and treatment of most patients with systemic corticosteroids leads to resolution of the disease. 
